# Influence of Silicone Rubber Coating on the Characteristics of Surface Streamer Discharge

**DOI:** 10.3390/polym13213784

**Published:** 2021-10-31

**Authors:** Xiaobo Meng, Liming Wang, Hongwei Mei, Chuyan Zhang

**Affiliations:** 1 School of Mechanical and Electrical Engineering, Guangzhou University, Guangzhou 510006, China; mengxb@gzhu.edu.cn; 2 Tsinghua Shenzhen International Graduate School, Tsinghua University, Shenzhen 518055, China; 3 School of Information Engineering, China University of Geosciences (Beijing), Beijing 100083, China; zcy@cugb.edu.cn

**Keywords:** surface streamer discharge, silicone rubber coating, three-electrode arrangement, thermally stimulated current method, surface properties

## Abstract

A pollution flashover along an insulation surface—a catastrophic accident in electrical power system—threatens the safe and reliable operation of a power grid. Silicone rubber coatings are applied to the surfaces of other insulation materials in order to improve the pollution flashover voltage of the insulation structure. It is generally believed that the hydrophobicity of the silicone rubber coating is key to blocking the physical process of pollution flashover, which prevents the formation of continuously wet pollution areas. However, it is unclear whether silicone rubber coating can suppress the generation of pre-discharges such as corona discharge and streamer discharge. In this research, the influence of silicone rubber coating on the characteristics of surface streamer discharge was researched in-depth. The streamer ‘stability’ propagation fields of the polymer are lower than that of the polymer with silicone rubber coating. The velocities of the streamer propagation along the polymer are higher than those along the polymer with silicone rubber coating. This indicates that the surface properties of the polymer with the silicone rubber coating are less favorable for streamer propagation than those of the polymer.

## 1. Introduction

Pollution flashover along the insulation surface occurs widely in electrical power systems, which threatens the safe and reliable operation of the power grid. The hydrophobicity of silicone rubber coating can prevent the formation of continuously wet pollution areas, and thus it can block the physical process of pollution flashover along the insulation surface. Therefore, silicone rubber coatings have typically been applied to the surfaces of other insulation materials in order to increase the pollution flashover voltage of the insulation structure.

In the long-term operation of an electrical power system, a partial pre-discharge may occur on the surface of the silicone rubber coating and cause it to gradually lose hydrophobicity. At the same time, a partial arc can also develop more easily due to the existence of a partial discharge, and then the pollution flashover voltage will decrease [1]. However, it is unclear whether the silicone rubber coating suppresses or promotes the generation of pre-discharges such as corona discharges and streamer discharges. Therefore, the influence of silicone rubber coating on the characteristics of partial pre-discharges needs to be researched in depth. It is necessary to find ways to suppress the partial pre-discharge on the surface of silicone rubber coating.

There have been many studies on the engineering applications of silicone rubber coating in electrical power systems, which have provided many theoretical bases for the engineering applications of silicone rubber coatings [2,3,4,5,6,7,8,9,10]. However, research on the characteristics of the partial discharge on the surfaces of silicone rubber coatings is rare. Streamer discharge is the most complex physical process in partial discharge, which develops into leader discharge and surface flashover within high-enough electric fields [11,12,13,14,15,16,17,18]. The streamer ‘stability’ propagation fields in air were lower than those on the insulation surface [13]. When there was streamer propagation along the insulation surface, there were ‘surface’ and ‘air’ components of the streamer discharge [14,15,16]. We have previously obtained photographs of the streamer discharge, observing the ‘surface’ component of the streamer propagated along the insulation surface having a higher velocity, and the ‘air’ component of streamer propagated in the air having a lower velocity [17]. In [17], the influence of the dielectric materials on the characteristics of the streamer discharge was also researched; the conclusion was that both the permittivity and the surface properties of dielectric materials affected the streamer discharge, which affected the subsequent flashover processes. Therefore, research on the characteristics of streamer propagation along the surface of the silicone rubber coating is conducive to a deeper understanding of the mechanism of partial discharge. If the partial arc discharge can be suppressed during the streamer propagation stage, the external insulation performances of silicone rubber coating will be greatly improved.

The paper [18] designed an experiment to describe the quantitative influence of permittivity and surface properties on the characteristics of streamer propagation along insulation surfaces. In this paper, a test of the characteristics of the streamer propagation along the polymer and the polymer with a silicone rubber coating was designed, which measured them using three photomultipliers and an ultraviolet camera. Because the silicone rubber coating was very thin, the overall permittivity of the polymer with the silicone rubber coating hardly changed. The differences between the streamer propagation along the polymer and the polymer with the silicone rubber coating were determined by comparing the characteristics of the surface streamer discharge from those materials. Not only could the test results be used as a verification of the previous test results in [18], but also the influence of a silicone rubber coating on the surface streamer propagation process was analyzed. In addition, the characteristics of the streamer propagation along the silicone rubber coatings produced by different manufacturers were compared, which provided a feasible method for evaluating the insulation performances of silicone rubber coatings.

## 2. Experiment Arrangement and Measurement System

**Figure 1:**
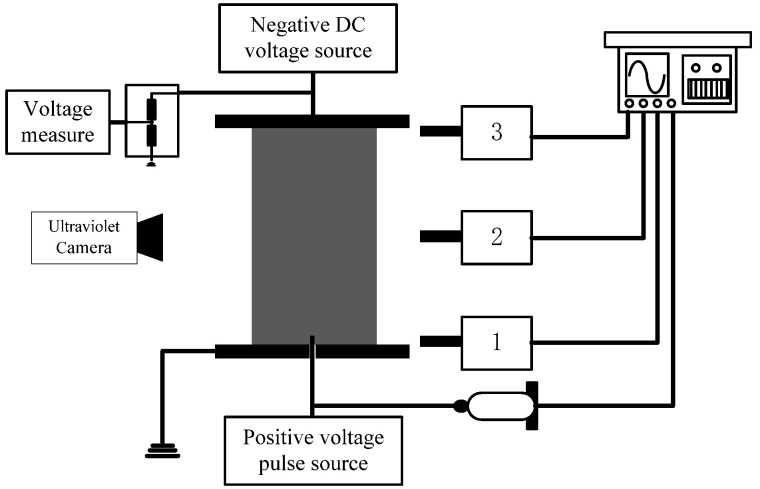
Schematic of the experiment arrangement and measurement equipment.

Figure 1 is a schematic diagram of the test equipment and measurement system used. Two flat electrodes and one needle electrode formed a three-electrode structure. The diameter of the parallel plates was 250 mm, and the distance between the upper and lower plates was 100 mm. The needle electrode was located at the circular hole (10 mm in diameter) in the center of the lower plates. The needle electrode was 2–3 mm above the plane of the lower plate, and was insulated from the lower plate. A negative DC voltage was applied to the upper plate, which was divided by a resistor divider, and then connected to a voltage measuring instrument via a coaxial cable. The lower plate was grounded. A square pulse voltage with adjustable amplitude and pulse width (1~6 kV, 100~250 ns) was applied to the needle electrode to trigger the positive polarity discharge. The square pulse voltage was divided by a high-voltage probe (Tektronix P6015A) that served as the trigger signal of a 4-channel 2 GHz oscilloscope (Agilent DSO7104A).

Three photomultipliers, each with a narrow slit (1 mm wide), were respectively directed at grazing incidence to the needle electrode, the middle position of the parallel plates, and the upper plate. The photomultiplier could monitor the development process of the streamer, because the head of the streamer would radiate photons into the space. The ‘DayCor@Superb’ UV imaging detector made by Ofil Corporation was used to take the photographs of the streamer discharge.

The polymer sheet used in the test was made of polyamide, and was placed vertically between two parallel plates. The polymer sheet was a square plate with a length of 100 mm, a width of 100 mm, and a thickness of 5 mm. The first polymer sheet was clean and had no silicone rubber coating, namely it was a polymer sheet. The second polymer sheet was coated with the first silicone rubber coating, namely Coating A. The last polymer sheet was coated with the second silicone rubber coating made by another manufacturer, namely Coating B. The permittivity of the polymer was 5, the permittivity of the first silicone rubber coating was 3.6, and the permittivity of the second silicone rubber coating was 3.8. The dielectric strength of Coating A was 22.2~22.8 kV/mm, and the dielectric strength of Coating B was 22.5~23.2 kV/mm. Their volume resistivity was 1.6 × 10^14^~1.8 × 10^14^ Ω m. For this study, the silicone rubber coatings were sprayed onto the surface of the polymer sheets, their thickness was 0.5 mm, and their surface drying time was 18–25 min. During the test, the indoor temperature was stable at about 25 °C, the relative humidity was maintained at about 65%, and the air pressure was the standard atmospheric pressure.

## 3. Experimental Results

### 3.1. Streamer Propagation Fields

**Figure 2:**
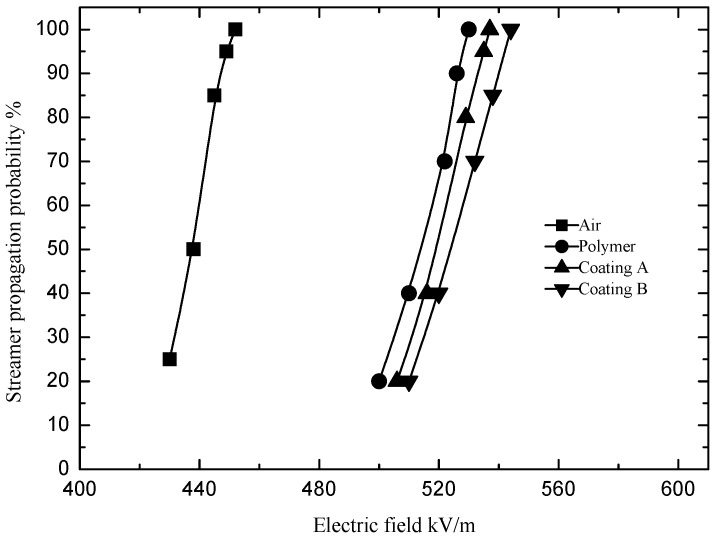
Relationship between the probability of streamer propagation and the guiding electric field.

Allen defined the applied electric field with a probability of 97.5% of the streamer propagating to the cathode plate as the streamer ‘stability’ propagation electric field *E*_st_ [13]. This definition was adopted in this study. The measurement method of the streamer stable propagation electric field was briefly as follows. The pulse voltage amplitude on the needle electrode was kept at a certain value *U*_pulse_, and the DC voltage *U*_app_ applied between the plates was increased gradually. The DC voltage between the two parallel plates gradually increased from 450 kV/m to 750 kV/m. At each DC voltage value *U*_app_, voltage pulses were applied to the needle electrode 20 times with a pulse interval of 20 s. That setting of the pulse interval was to ensure that the remaining ions from the previous streamer discharge were fully diffused. As the voltage between the two parallel plates gradually increased, the propagation probability of the streamer gradually increased from 0% to 100%. The streamer propagation probability and the applied electric field satisfied the Gaussian distribution function as shown in Figure 2. The Gaussian distribution function (1) was used to fit the statistical distribution curve of the streamer propagation probability with the electric field.

(1) $$y=y_{0}+\frac{A}{w\sqrt{\pi /2}}e^{-2\frac{{\left(E-E_{c}\right)}^{2}}{w^{2}}}$$

In Formula (1), *E* is the electric field, *E*_c_ is the mean value of the electric field; *w* is the variance; *y* is the streamer propagation probability; *A* and *y*_0_ are undetermined coefficients.

**Figure 3:**
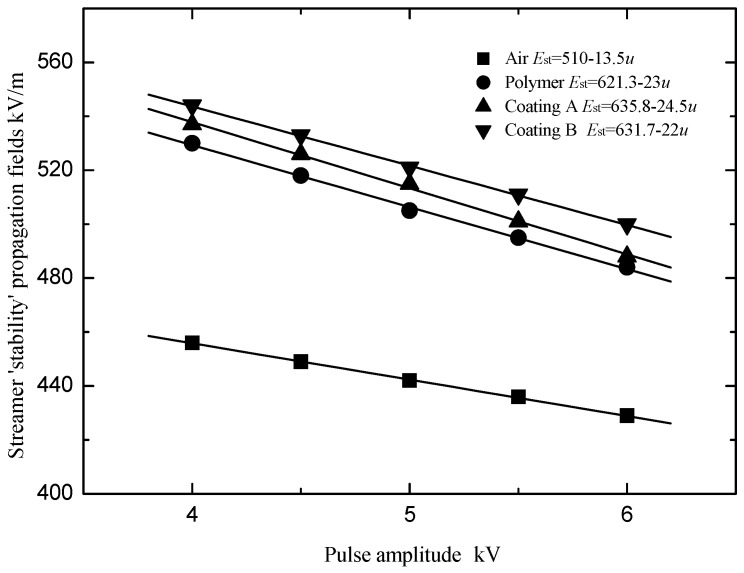
Relationship between the streamer ‘stability’ propagation fields and the pulse amplitude.

Then, the streamer ‘stability’ propagation fields *E*_st_ (streamer propagation probability of 97.5%) were obtained as shown in Figure 3. It was found that the streamer ‘stability’ propagation fields decreased linearly with the increase in the applied pulse amplitude. The reason is that the energy initially obtained by the streamer from the applied pulse increases with its amplitude, and the subsequent streamer propagation becomes easier. In addition, it can be seen that the streamer ‘stability’ propagation fields with the polymer with the silicone rubber coating are stronger than those with the polymer. Furthermore, the electric fields for the streamer stable propagation along the surface of the different silicone rubber coatings are quite different.

The fitting Formula (2) was used in Figure 3 to fit the curve. *E*_st_ is the streamer ‘stability’ propagation field, *E*_0_ is the streamer stable propagation electric field when the pulse amplitude is 0 kV, u is the pulse amplitude, and *α* is an undetermined coefficient.


(2) $$E_{st}=E_{0}-\alpha u\;(kV/m)$$

### 3.2. Light Emission

A large number of photons are generated during the process of streamer discharge. Some of them participate in the photoionization in the discharge area, while others escape to the outside. The photoionization plays a vital role in the generation and development of streamers. The secondary electron avalanches generated by the photoionization in front of the streamer head supply the streamer discharge with positive and negative charges, and then the streamer channel moves forward. 

**Figure 4:**
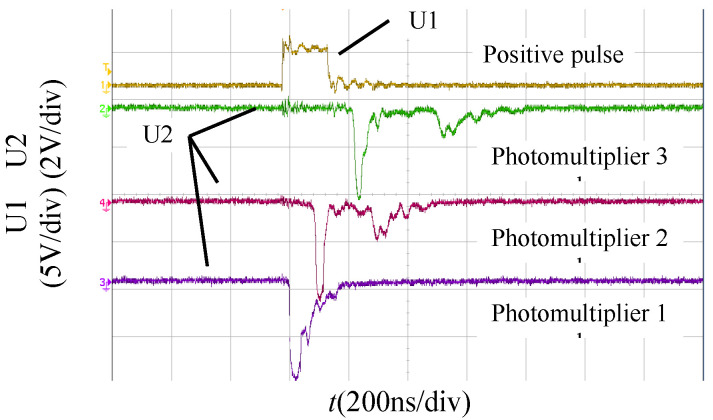
Typical signals from the photomultiplier monitoring the streamer propagation along the surface of the polymer.

Based on the physical phenomenon of photons being emitted outward during the streamer discharge, the photomultipliers and UV imaging detector were used to observe the process of the streamer discharge. In our previous articles [17,18], we found that there were ‘surface’ and ‘air’ components of the streamer discharge when streamer propagation occurred along the insulation surface. The ‘surface’ components of the streamers propagated along the insulation surfaces at a higher velocity, and the ‘air’ components of the streamers propagated in the air at a lower velocity. However, there were only ‘air’ components of the streamer discharges when the streamers propagated in air alone. The same conclusion was reached in this article. The photomultiplier detected two peaks of light at the cathode plate when the streamers propagated along either the polymer or the polymer with the silicone rubber coating as shown in Figure 4. Therefore, the ‘surface’ and ‘air’ components of the streamer discharges also occurred along both the polymer and the polymer with the silicone rubber coating. The ‘surface’ components of the streamers had higher velocities and their propagation paths lay along the insulation surfaces. The ‘air’ components of the streamers had lower velocities and their propagation paths were in the air.

**Figure 5:**
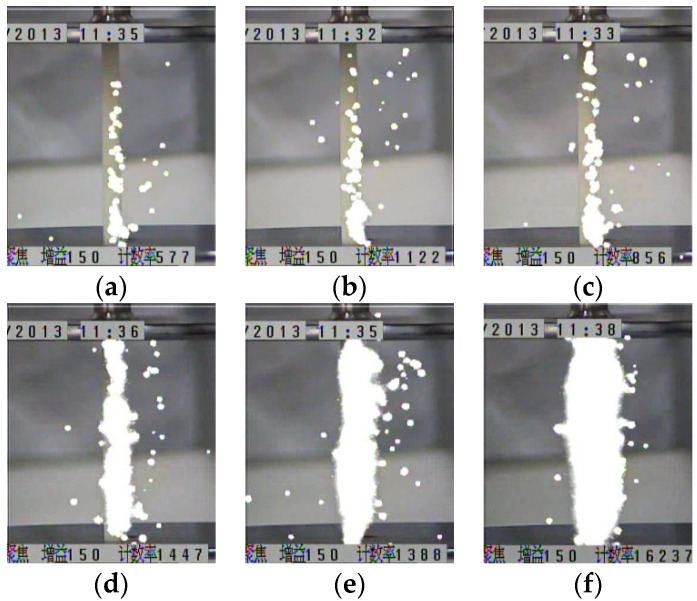
Streamer propagation photographs for the polymer. (**a**) 500 kV/m, (**b**) 530 kV/m, (**c**) 550 kV/m (**d**) 590 kV/m, (**e**) 620 kV/m, (**f**) 660 kV/m.

**Figure 6:**
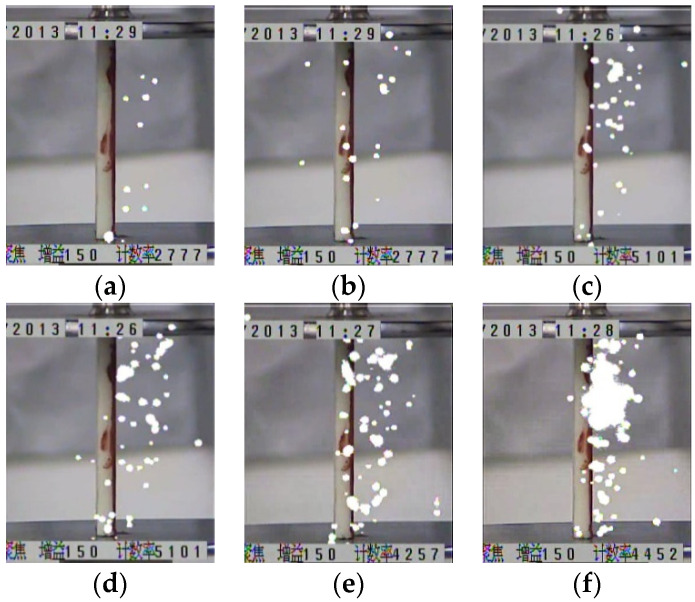
Streamer propagation photographs for the polymer with the silicone rubber coating. (**a**) 510 kV/m, (**b**) 540 kV/m, (**c**) 560 kV/m, (**d**) 590 kV/m, (**e**) 630 kV/m, (**f**) 660 kV/m.

The UV imaging detector was used to take the photographs of the streamer discharges. The light emitted from a single propagation process of a streamer was able to be recorded in a clear image. The white spots on each image are the signal displayed by the light emitted from a streamer discharge. Figure 5 and Figure 6 show the streamer propagation photographs for the polymer and the polymer with the silicone rubber coating. 

It can be observed that the ‘surface’ component of a streamer propagates along the insulation surface, while the ‘air’ component of a streamer propagated in the air and was away from the insulation surface. Within the same electric field, the luminous intensity of the streamer propagation along the polymer was greater than that along the polymer with the silicone rubber coating. Furthermore, the luminous intensity of the streamer propagation along the polymer with the different silicone rubber coatings was also different. It was determined that the luminous intensity of a streamer was closely related to the subsequent photoionization. The stronger the luminous intensity of a streamer was, the more intense the subsequent photoionization would be, and it would promote the development of the subsequent streamer. This also explains that the electric fields required for the streamer stable propagation along the polymer with the silicone rubber coating were greater than that along the polymer.

### 3.3. Streamer Propagation Velocity

**Figure 7:**
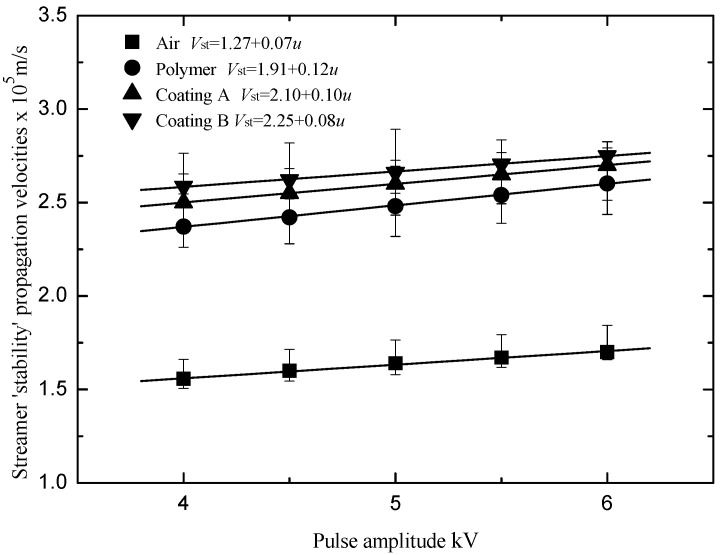
Relationship between the streamer ‘stability’ propagation velocities and the pulse amplitudes.

The propagation velocity of the streamers was calculated by the ratio of the vertical distance between the three photomultipliers and the time difference ΔT between the starting points of the rising edge of the light signals from the three photomultipliers (Figure 4). The streamer ‘stability’ propagation velocity *V*_st_ was defined as the streamer propagation velocity within the ‘stability’ electric field. Figure 7 shows the relationship between the streamer ‘stability’ propagation velocities and the pulse amplitude. The ‘stability’ velocities of the streamer propagation along the surface of the polymer with a coating were linearly related to the pulse amplitude. For Figure 7, Equation (3), which relates the streamer ‘stability’ propagation velocities to the pulse amplitude, was used to fit the curves. *u* is the pulse amplitude, kV; *V*_0_ is the streamer stable propagation velocity when the pulse amplitude is 0 kV, 10^5^ m/s; *β* is the undetermined coefficient.

(3) $$V_{\mathrm{st}}=V_{0}+\beta u$$

**Figure 8:**
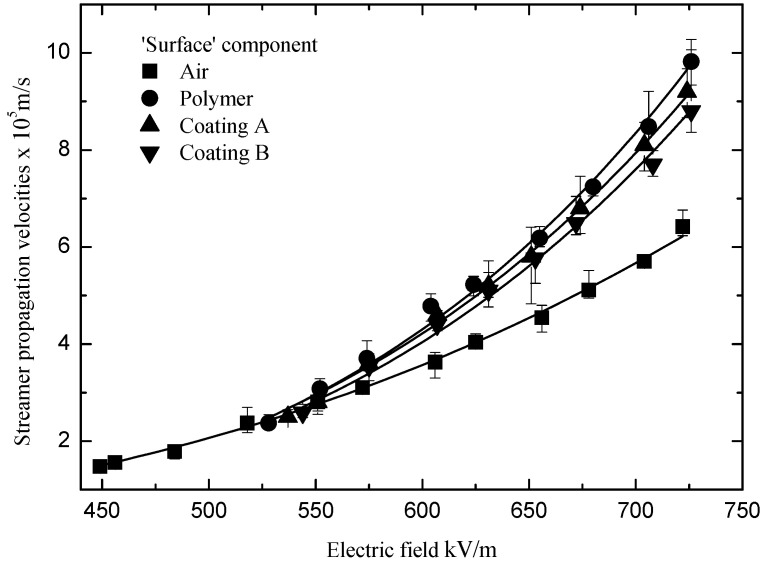
Velocities of the ‘surface’ components under the varied electric fields.

**Figure 9:**
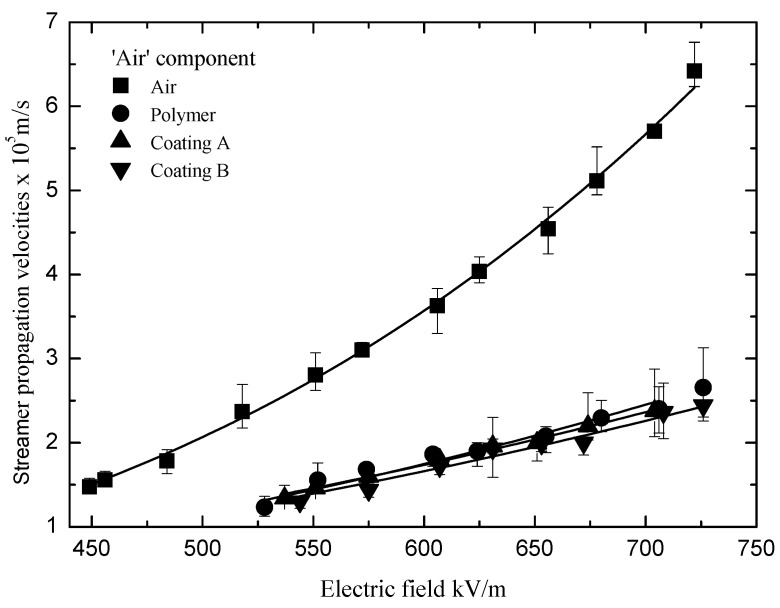
Velocities of the ‘air’ components under the varied electric fields.

The ‘surface’ and ‘air’ components of the streamers occurred when the applied electric fields were larger than the streamer ‘stability’ propagation fields. The velocities of the ‘surface’ and ‘slow’ components under the varied electric fields are displayed in Figure 8 and Figure 9, respectively. Equation (4) was used to draw the fitting curves in Figure 8 and Figure 9. *E*_st_ and *V*_st_ come from Equations (2) and (3), and *n* and *γ* are the undetermined coefficients listed in Table 1.

(4) $$V_{s}=V_{\mathrm{st}}{\left(\frac{E}{E_{st}}\left(1+\gamma \right)\right)}^{n}$$

*E*_st_ and *V*_st_ in Equation (4) were replaced by Equations (2) and (3) to become Equation (5). It describes the streamer propagation velocities under any pulse amplitude and applied electric field.

(5) $$V_{s}=\left(V_{0}+\beta u\right){\left(\frac{E\left(1+\gamma \right)}{E_{0}-\alpha u}\right)}^{n}$$

In Figure 8 and Figure 9, it can be seen that the velocities of the ‘surface’ components of the streamers were higher than those in the air alone, and they increased with the applied electric field significantly. In contrast, the velocities of the ‘air’ components of the streamers were lower than those in the air alone, and they increased with the applied electric field slowly. It can be explained that the electric field in the head of the ‘air’ component of a streamer is suppressed by the charge in the head of the ‘surface’ component. Furthermore, it can be seen that the velocities of the ‘surface’ components of the streamer decreased after the silicone rubber coating was applied to the polymer. In addition, the velocities of the ‘surface’ components of the streamers propagating along the different silicone rubber coatings were also different. However, the differences between the velocities of the ‘air’ components of the streamers propagating along the different insulation surfaces were relatively small.

## 4. Discussion

### 4.1. Permittivity

**Figure 10:**
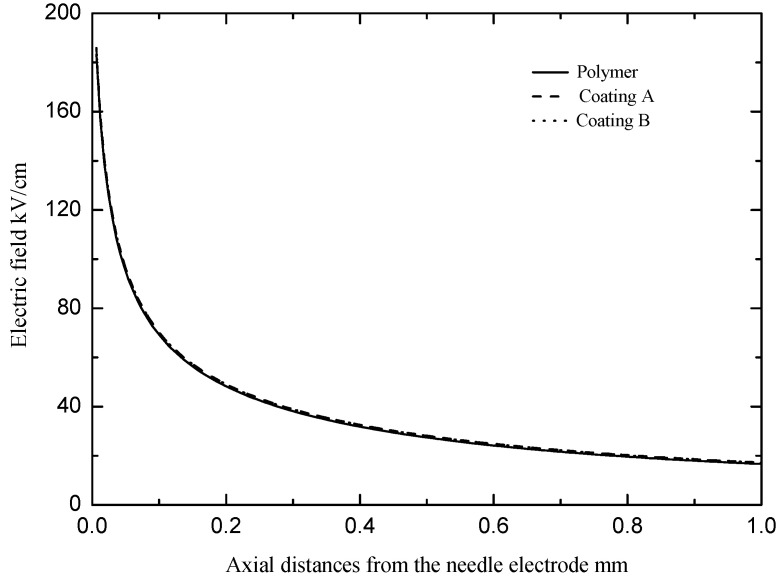
Variation of the electric field from the needle electrode up to 1 mm at axial distances.

The main factors that affected the characteristics of the streamer propagation along the insulation surfaces were the permittivity and surface properties (the attachment of the charge to the surface, photoemission of secondary electrons from the surface, etc.) [18]. First, the influence of the silicone rubber coating on the permittivity of the polymer sheet was analyzed. Figure 10 shows the variation of the electric field from the needle electrode up to 1 mm along the insulation surface. The thickness of the silicone rubber coating was considered to be 0.5 mm. It can be seen that the electric fields from the needle electrode up to 1 mm along both the polymer and the polymer with the silicone rubber coating were basically the same, but the electric field along the polymer with the silicone rubber coating was slightly strengthened. The permittivity of the silicone rubber coating was smaller than that of the polymer. After the silicone rubber coating was applied to the polymer, the volume of the polymer sheet with the silicone rubber coating became larger than that of the polymer sheet. The overall capacitance (permittivity) increased, so the electric field along the polymer sheet with the silicone rubber coating increased. However, the silicone rubber coating only had a small increase in the electric field at the tip of the needle electrode, which indicates that the silicone rubber coating produced a small change in the overall permittivity (capacitance) of the polymer sheet. Therefore, the change in the overall permittivity caused by the silicone rubber coating had a tiny impact on the electric field distribution in the gap. 

**Figure 11:**
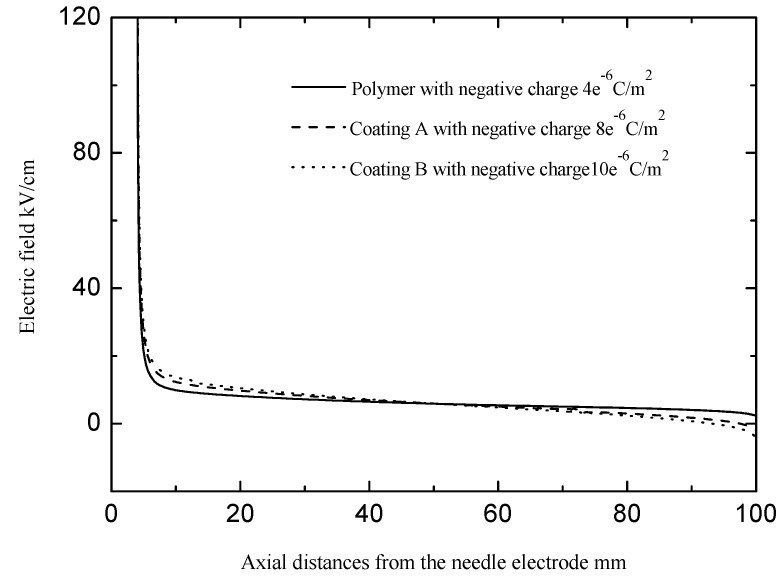
Variation of the electric field in the gap at axial distances.

With the increase in the permittivity, the charge (ions or electrons) accumulated on the insulation surface increased [19,20,21]. On the one hand, there were many negative charges accumulated on the insulation surface with the negative direct voltage applied to the cathode plane [22,23,24]. Those would have reduced the electric fields in the latter half of the sheet as shown in Figure 11. The negative charges on the surface weakened the electric fields in the latter half of the sheet. Hence, the streamer propagation along the sheet with the larger permittivity required higher electric fields [17]. On the other hand, the sheet with the larger permittivity would have attached more positive charges in the streamer. The ionization efficiency at the head of the streamer would have weakened, which made the streamer propagation difficult and required high electric fields. Because the silicone rubber coating made the overall permittivity (capacitance) of the polymer sheet slightly increase, the silicone rubber coating caused a slight increase in the positive charge in the streamers attached to the surface, which would have suppressed the development of those streamers to a certain extent. However, the stable streamer propagation fields of the polymer sheet and the polymer sheet with the silicone rubber coating displayed a large difference. The change in the overall permittivity caused by the silicone rubber coating should not have caused such a large difference. It must have been caused by the change in the surface properties of the polymer sheet after spraying the silicone rubber coating.

### 4.2. Surface Properties

When the silicone rubber coating was applied to the polymer sheet, the surface condition changed greatly. The roughness of the materials was measured using the roughness gauge. The roughness of the three materials was as follows: Coating B was the largest (Ra of 0.93 μm), Coating A was second (Ra of 0.76 μm), and the polymer was the smallest (Ra of 0.65 μm). The larger the surface roughness of the material was, the more serious the accumulation of the surface charge was [21]. Therefore, the negative charges accumulated on the surface increased with the increases in the surface roughness, which had two influences on the streamer discharges. One is that the electric fields in the latter half of the sheet weakened due to the negative surface charges; the other is that the ionization efficiency at the head of the streamers weakened due to the attachment of the positive charges in the streamer to the surface. It was more difficult for the streamers to propagate along the sheet with the higher surface roughness. That is the reason why the streamer ‘stability’ propagation fields for the polymer with the silicone rubber coatings were larger than those for the polymer. In addition, the electric fields for the streamer stable propagations along Coating B were larger than those along Coating A at the different surface roughness levels.

**Figure 12:**
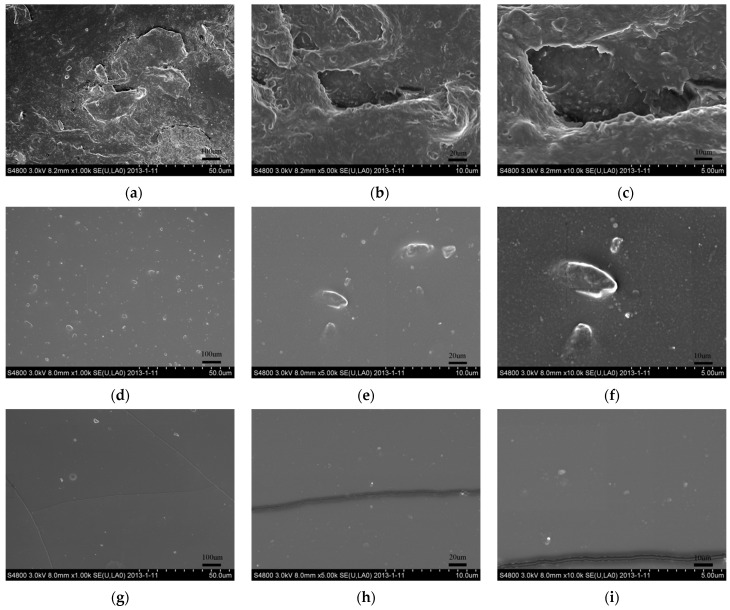
SEM figures of the insulation surfaces. (**a**–**c**) Polymer. (**d**–**f**) Coating A. (**g**–**i**) Coating B.

Figure 12 shows the surface conditions of the three insulation materials measured by the scanning electron microscope. It can be seen that the surface of the polymer had more microporous defects than the polymer with the silicone rubber coatings. The trap charges (nC) and trap levels (eV) of the three insulation materials were tested using the method of the thermally stimulated current (TSC). The trap charges on the polymer surface were greater than those on the silicone rubber coatings. The microporous defects on the insulation surfaces could reflect the surface trap distributions [21]. The trap charges on the insulation surface decreased with the decreases in the microporous defects. Hence, the results of the SEM figures and the TSC test corroborate each other.

The traps that had low trap levels are named “shallow traps”. In Table 2, the shallow traps on the polymer surface were greater than those on the silicone rubber coatings. The photoemission of secondary electrons from the surfaces can be described as follows: the collisions of the high-energy photons detach the trap charges from the insulation surface and produce many high-energy secondary electrons, which then promote the development of the streamers [13,14]. Under the collision by the high-energy photons, a shallow trap emits high-energy secondary electrons more easily. The reason why the streamer propagation along the silicone rubber coating is more difficult than that along the polymer is that the photoemission of secondary electrons from the silicone rubber coating is weaker. The shallow traps on the Coating A were greater than those on Coating B. Therefore, the stronger photoemission of secondary electrons from Coating A led to the streamer propagation more easily, which is consistent with the test results.

In a word, the polymer surface was more favorable for the streamer propagation than the silicone rubber coating surface from the perspectives of both the surface roughness and surface trap. The electric fields required for the streamer stable propagation along the silicone rubber coating were larger than that required for the streamer stable propagation along the polymer surface. The velocities of the streamer propagation along the silicone rubber coating were lower than that along the polymer surface under the same electric fields. From the perspective of surface properties, it was also a good explanation of the differences between the streamer stable propagation fields and velocities along the different silicone rubber coatings produced by the different manufacturers. There were differences in the characteristics of the streamer propagation along the different silicone rubber coatings, which indicates there are large differences in the insulation properties of the silicone rubber coatings belonging to different manufacturers. These differences can be found by measuring the characteristics of the streamer discharge. Therefore, tests of the streamer discharge can be used to evaluate the insulation properties of silicone rubber coatings.

The test results of the characteristics of streamer propagation along the different material surfaces have also taught us many things. Higher permittivity in a material is unfavorable for streamer propagation along it. Hence, materials with higher permittivity can be chosen to suppress the pre-discharge in some conditions of electrical power systems. It is difficult for streamer propagation to occur along materials with higher macroscopic surface roughness, so insulation surfaces can be made rougher to reduce pre-discharge in electrical power systems. The microporous defects on the insulation surface can affect the streamer propagation to a great extent. In the factory, more nanomaterials can be applied to insulation materials to fill the microporous defects on the insulation surface, thereby the pre-discharge will be prevented by the technology of reducing microporous defects. These results provide a theoretical basis for promoting the application of the nanomaterials.

## 5. Conclusions

The streamer ‘stability’ propagation fields for the polymer, Coating A and Coating B were 528 kV/m, 537 kV/m and 544 kV/m, respectively. The velocities of the ‘surface’ components of the streamer stable propagation along the polymer, Coating A and Coating B were 2.37 × 10^5^ m/s, 2.50 × 10^5^ m/s and 2.58 × 10^5^ m/s, respectively. The velocities of the ‘air’ component of the streamer stable propagation along the polymer, Coating A and Coating B are 1.23 × 10^5^ m/s, 1.34 × 10^5^ m/s and 1.30 × 10^5^ m/s, respectively. The streamer ‘stability’ propagation fields for the polymer were lower than those for the polymer with the silicone rubber coatings. Within the same electric fields, the velocities of streamer propagation along the polymer were higher than those along the polymer with the silicone rubber coatings.

Higher permittivity in a material was unfavorable for streamer propagation along it. The effects of permittivity on electric field distortion in front of needle tip, the effects of the surface charge accumulation before the development of a streamer on the distortion of the electric field in the gap and the effects of the charge attachment to the surface during the development of streamer were analyzed to determine the reason.

It is difficult for streamer propagation to occur along materials with higher macroscopic surface roughness. The effects of the surface charge accumulations before the development of streamers on the distortions of the electric field in the gap and the effects of the charge attachment to the surface during the development of streamers were also analyzed to determine the reason.

The streamer propagation along materials with more microporous defects on the insulation surface was easier. The reason is that the photoemission of secondary electrons from the surface increased with increased microporous defects, which would have promoted the development of the streamer.

There are large differences in the characteristics of surface streamers along the different silicone rubber coatings. Testing the streamer discharge can be used to evaluate the insulation properties of the silicone rubber coatings produced by the different manufacturers.

## Figures and Tables

**Table 1:** Corresponding parameters in Equation (3).

Material	*E*_st_	‘Surface’ Component			‘Air’ Component		
		*V*_st_	*γ* × 100	*n*	*V*_st_	*γ* × 100	*n*
Air	456	1.56	0.22	3	1.56	0.23	3
Polymer sheet	528	2.37	0.15	4.3	1.23	3.24	2.2
Coating A	537	2.50	1.69	4.1	1.34	0.75	2.1
Coating B	544	2.58	1.16	4.1	1.3	2.48	2.2

**Table 2:** Trap parameters of the dielectric materials measured by TSC test.

Parameter	Polymer	Coating A	Coating B
Current peak (PA)	1050	165	138
Trap charge (nC)	1879	246	225
Trap level (eV)	0.38	0.45	0.49

## Data Availability

Not applicable.
